# Severe Ocular Trauma and the Race Against Time in its Management: A Case Series

**DOI:** 10.7759/cureus.32676

**Published:** 2022-12-19

**Authors:** Devansh Satyawali, Vivekanand Satyawali, Shanti Pandey

**Affiliations:** 1 Medical School, Vardhman Mahavir Medical College and Safdarjung Hospital, Delhi, IND; 2 Internal Medicine, Government Medical College, Haldwani, Haldwani, IND; 3 Department of Ophthalmology, Government Doon Medical College, Dehradun, IND

**Keywords:** orbital trauma, vision loss, trauma, globe luxation, intraorbital foreign body, ocular trauma

## Abstract

Ocular injury is one of the most important causes of vision loss. We present a case series of different ocular trauma cases with rare presentations which presented a great challenge to emergency management. The management of ocular injuries is a fight against time, with not only the vision but also at times present with the life of patients at risk. These thus require timely diagnosis.

The first case involves a sharp penetrating injury with a metallic foreign body in the orbit. The second case is of a retained intraorbital foreign body secondary to a gunshot injury. The third case is of traumatic globe luxation secondary to a blunt injury. Lastly, the fourth case is of an animal bite with a lacerated wound. All the patients in the reported cases presented to the casualty of the Government Medical College, Haldwani. We believe this case series will add to the literature and help ophthalmologists gain experience dealing with such cases.

## Introduction

Blunt eye trauma can cause a variety of intrinsic eye injuries. Open- and closed-globe injuries can develop from blunt trauma. Contusions and lamellar lacerations are two types of closed-globe injuries. Laceration and globe rupture are two types of open globe injuries. Laceration can be caused by a penetrating injury, a perforation injury, or an intraocular foreign body (IOFB). A direct strike to the eyeball or inadvertent physical trauma might cause harm. Approximately 1.6 million people have lost their vision due to ocular injuries [1]. The incidence of globe injuries is estimated to be 3.5 eye injuries per 100,000 people, with males accounting for roughly 80% of open globe injuries. Sharp items immediately entering the globe cause more injuries in the paediatric population (e.g., writing utensils, scissors, or knives) [2]. Ocular trauma presents a great challenge for diagnosis and management. After determining that the patient is stable and that any other major nonocular injuries have been handled, a detailed medical/surgical history is collected, followed by a more focused ocular history. Prior surgery, trauma, and any previously existing eye illness are all important considerations. A thorough assessment is carried out in a methodical and logical manner, commencing with a thorough external inspection. Each eye's visual acuity is evaluated separately. Testing for a relative afferent pupillary deficiency, completing gross confrontational visual field testing, identifying any relative difference in subjective brightness perception, and examining colour vision are all used to evaluate optic nerve function. If necessary, the intraocular pressure (IOP) is measured and a detailed slit lamp examination with dilated indirect ophthalmoscopy is conducted. A quick penlight check will typically detect obvious open globe injury. Patients who are uncooperative should be assessed under anaesthesia in a restricted, monitored setting with skilled critical care workers. Auxiliary testing can provide additional information (primarily CT and ultrasonography). If the combination of clinical symptoms and ancillary tests is still inconclusive, formal exploration in the operating room under anaesthesia is advised [3].

We provide a case series of four ocular trauma cases with unusual presentations that posed a great challenge to emergency management. The treatment of ocular injuries involves a race against time, with not only the patients' vision at stake but even their lives at times. As a result, prompt diagnosis is required.

## Case presentation

Case 1

**Figure 1:**
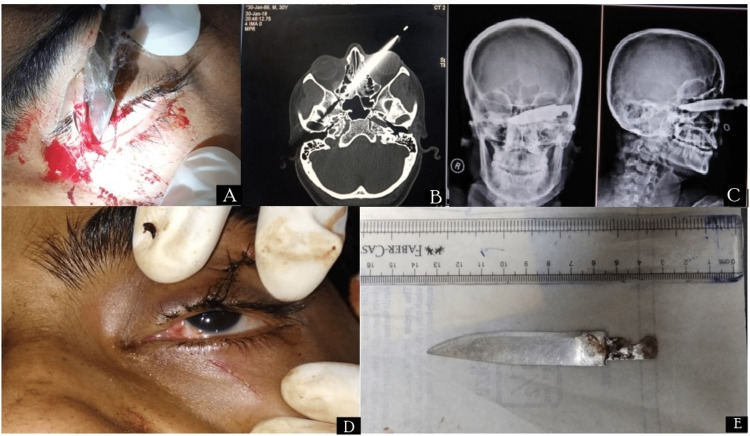
Case 1 (A) Image of stab injury with a knife in medial canthus; (B) non-contrast computed tomography (NCCT) of orbit showing foreign body (FB); (C) PA/lateral view X-ray of skull showing FB; (D) Image of patient post-FB removal; (E) FB shown with scale.

A 30-year-old female presented to the casualty department with an alleged history of assault with a sharp metallic object two hours previously, with the chief complaint of left eye pain and nasal bleeding. On ophthalmological examination visual acuity was 6/6 in the right eye and finger counting 3m in the left eye; a sharp metallic foreign body was present at the medial canthus penetrating into the left orbital medial wall through the upper eyelid, sparing the globe. Extraocular movements (EOM) were restricted in the left eye. NCCT (non-contrast computed tomography) axial scans of the orbit showed a foreign body penetrating the medial wall of the left orbit deep into the left nasal cavity involving the nasal septum and extending up to right nasal cavity and sparing the globe and medial rectus muscle (Figure 1). Detailed ENT evaluation was done by an otorhinolaryngologist. The patient was planned for foreign body removal under general anaesthesia. A 10 cm long metallic foreign body was removed, and a detailed examination of the globe was performed to look for any other injuries. A conjunctival laceration was left as it was and the lid laceration was repaired with 6.0 silk. Nasal packing was applied (Figure 1). Post-operative IV antibiotics and oral non-steroidal anti-inflammatory medications were given for five days. In addition, topical antibiotic drops, and ointment was prescribed for local application. Post-operative recovery was uneventful. The best corrected visual acuity (BCVA) post-operation in Week 1 was 6/12 OD and 6/6 OS. Extraocular motility was full. Dilated fundus examination was within normal limits (WNL).

Case 2

**Figure 2:**
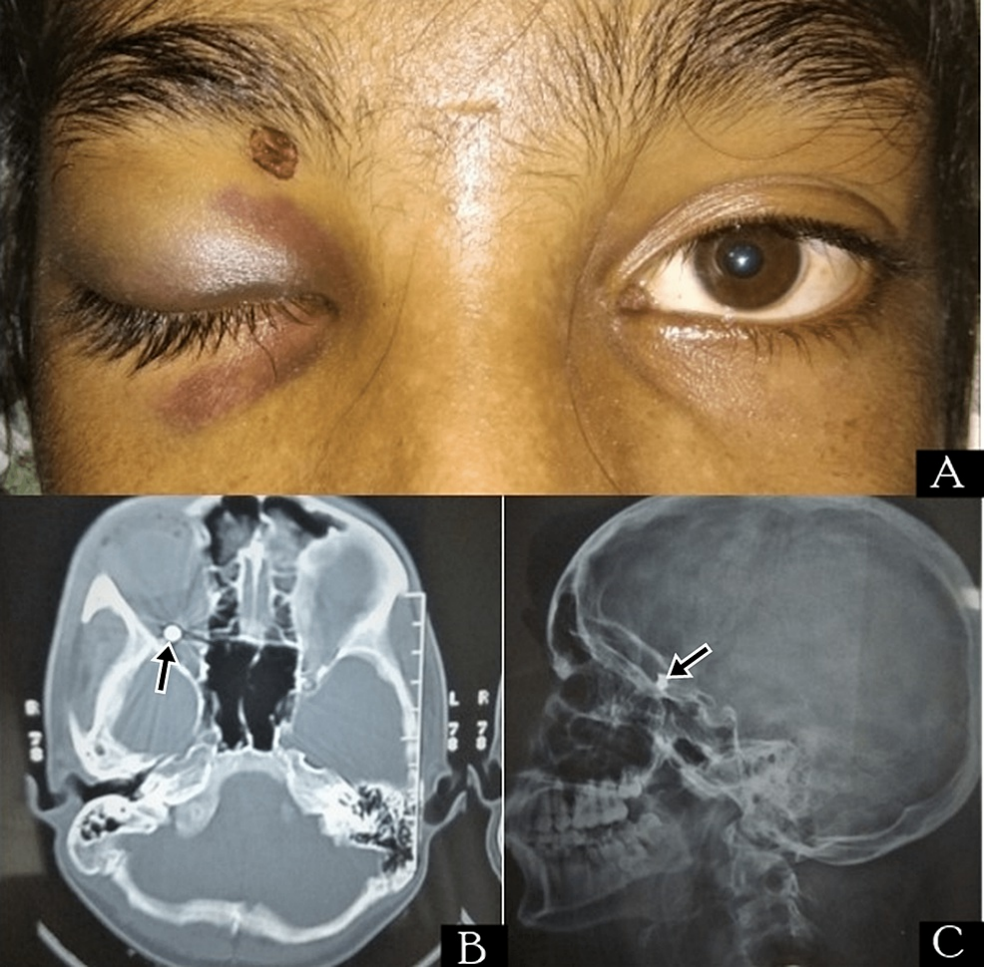
Case 2 (A) Right eye ecchymosis, ptosis and small right upper lid wound; (B) X-ray showing foreign body (FB) in intraconal space; (C) non-contrast computed tomography (NCCT) showing FB in the orbital apex.

A 15-year-old male presented with an alleged history of assault two days back, with chief complaints of painful swelling of the right eye. On examination BCVA of both eyes was 6/6. Lid edema and periorbital ecchymosis with severe ptosis were present in the right eye with a small wound around 2 mm over the right upper eyelid near the orbital margin (Figure 2). EOMs were intact. Dilated fundus examination was WNL. IOP was 12mmHg and 13 mmHg in right and left eyes respectively. AP view and lateral view X-rays were performed to rule out any orbital bony injury. A metallic foreign body was found in intraconal space. NCCT scan was performed to localize the foreign body which was found to be lodged at the orbital apex. The patient was managed conservatively on oral antibiotics and oral non-steroidal anti-inflammatory drugs.

Case 3

**Figure 3:**
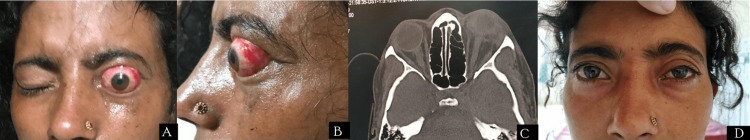
Case 3 (A) Left eye proptosis; (B) Lateral view showing axial proptosis (FB); (C) Non-contrast computed tomography (NCCT) showing axial proptosis and straightening of optic nerve; (D) Post-treatment pictures

A 36-year-old female came to the casualty department, with an alleged history of falls from height, with blunt injury to her left eye. Chief complaints were sudden bulging of the left eye, diminution of vision, and pain in the left eye. On examination visual acuity in the right eye was 6/6 and 6/36 in the left eye. The left eye had proptosis with retraction and rolling in both upper and lower lids with marked congestion of bulbar conjunctiva, with exposure keratopathy, the pupil was round, regular, and reactive to light. Dilated fundus examination was within normal limits. EOM was -4 in all gazes (Figure 3). Intraocular pressure in the right eye was 12mmHg and in the left eye was 16mmHg.

NCCT orbit was done to rule out any retroorbital hematoma or mass. It showed axial proptosis and straightening of the optic nerve of the left eye. Diagnosis of traumatic luxation of the globe was made. It was reduced with gentle manipulation under sedation. The eye was patched overnight. Post-operative oral anti-inflammatory and topical lubricants were given. The patient fully recovered, with full ocular motility (Figure 3). BCVA was 6/6 in both eyes.

Case 4

**Figure 4:**
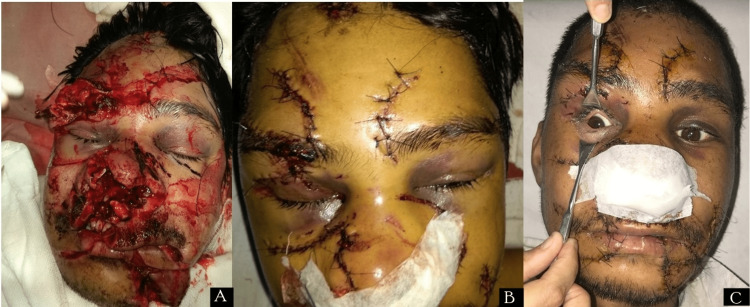
Case 4 (A) Deep and dirty lacerated wound over right eyebrow; (B) Wound after debridement and primary closure (FB); (C) Post-operative Day 7

A 45-year-old male presented to the emergency department with an alleged history of animal bite (bear bite). He has chief complaints of painful bleeding and lacerated wounds over his face. On examination, a deep dirty lacerated wound was present over the right eyebrow extending up to the forehead, with periorbital ecchymosis in both eyes with lid oedema (Figure 4A). BCVA in both eyes was 6/6. Pupils were round, regular, and reactive. EOM was intact in all gazes. The wound was lavaged with copious normal saline and 5% povidone-iodine solution, the wound was debrided, and primary closure of the wound was done with Vicryl 6.0 and silk 5.0 sutures in two layers (Figure 4B). Post-exposure anti-rabies and tetanus toxoid prophylaxis, immunoglobulins and anti-rabies vaccination were given according to schedule. The patient was kept on IV antibiotics and local wound care was done regularly. On post-operative Day 7, the lid oedema decreased, and wound healing occurred (Figure 4C).

## Discussion

Ocular trauma presenting to the ER presents a diagnostic and management challenge with a war against time to save the patient's life, their vision, and to preserve the aesthetics of the eye area. Penetrating ocular injuries cause significant mortality and morbidity in patients. Such injuries have the possibility of significant life-threatening non-ocular or orbital injuries. So, the assessment of vital signs and neurological function should be prioritized over the ophthalmological examination. Most ocular injuries cause direct and indirect injury to the globe and extraocular structures. Assessment of vision in threatening injuries should be done first, to rule out globe rupture and later, the orbital and adnexal injury should be assessed [4]. Patients should also be examined for the presence of injury to the sino-cranial compartment. Thus, making the management of such cases, very challenging even for the most experienced surgeons.

Detailed history taking and understating the mode and object of injury is the first step towards planning for the most effective management. Nature of foreign body and locating it with the help of diagnostic imaging is needed in almost every case. Orbital imaging can define the location and extent of injuries. Non-contrast computed tomography is the modality of choice. If a foreign body is suspected, a thin 1-2mm slice axial scan is preferable. If the foreign body has penetrated the intracranial space through the orbit, urgent neurosurgical consultation is required. If adjacent paranasal sinuses are involved, the surgical management is done in conjunction with an otorhinolaryngologist [5]. The foreign bodies that are inert, located deep, and not causing any functional deficit, infection or inflammation are the most difficult to treat as they cause a diagnostic dilemma; based on the literature available, such foreign bodies are managed conservatively [6-7]. However, this cannot be generalized and does not hold true for intravitreal foreign bodies.

Traumatic globe luxation is a rare presentation, which is usually associated with blunt trauma and orbital fracture. It occurs when the equator of the eye crosses the lid aperture due to a sudden increase in orbital pressure it also moves further with the contraction of the orbicularis oculi muscle which pushes the eye forward [8]. It also has been reported in some cases of floppy eyelid syndrome [8]. Traumatic globe luxation is associated with decelerating injury [9]. In such cases, detailed history taking and the mechanism of injury are important. A thorough clinical examination should be conducted as well as an assessment of vision, pupillary reaction, looking for exposure keratopathy, and fundus examination to look for the optic nerve head. The role of imaging is important in blunt injuries to evaluate orbital fractures, retroorbital hematoma, and optic nerve avulsion. Early recognition and timely reduction of the luxation is necessary to save the vision and to prevent further complications as any delay in treatment may cause optic nerve ischemia and extraocular muscle ischemia due to excessive stretching of tissues.

Animal bites are most frequently faced in the emergency department. The most commonly affected area in animal bites is the face and ocular adnexa. The reconstruction of ocular adnexa is important in both the short term and the long term. Proper wound care, salvaging the soft tissue, and repair of a wound are essential. Prevention of wound infection and tetanus, and anti-rabies vaccination is to be kept in mind at the same time [10]. Primary immediate closure of wounds is recommended for facial injuries [11]. Aggressive wound care with copious saline and betadine solution and primary wound closure with intravenous antibiotics play essential roles in managing such cases. Primary immediate closure of the wound is recommended for facial injuries [11]. Primary closure of wounds results in good postoperative outcomes [12]. Sometimes animal bites are associated with facial and orbital fractures, so baseline radiological investigation must also be done to rule out any fracture. Cosmetic correction must also be kept in mind while repairing as patients may need repeated surgical procedures.

## Conclusions

Ocular trauma presents a race against time and requires a detailed history, clinical examination, and radiological investigation for diagnosis and treatment of such cases. Early diagnosis and treatment by an ophthalmologist can improve the prognosis and help salvage vision. The prognosis is determined by various factors, including the time taken for the start of treatment, the degree of the injury, and the type of eye injury.

## References

[1] Négrel AD, Thylefors B (1998). The global impact of eye injuries. Ophthalmic Epidemiol.

[2] Mohseni M, Blair K, Gurnani B (2022). Blunt Eye Trauma. https://www.ncbi.nlm.nih.gov/books/NBK470379/.

[3] Harlan JB Jr, Pieramici DJ (2002). Evaluation of patients with ocular trauma. Ophthalmol Clin North Am.

[4] Mutie D, Mwangi N (2015). Assessing an eye injury patient. Community Eye Health.

[5] Albert Jackobic Principles and practice of ophthalmology, 3rd Edition, 2000 2000 (2008). Penetrating eyelid and orbital trauma. Albert Jakobiec's Principles and Practice of Ophthalmology, 3rd ed..

[6] Ho VH, Wilson MW, Fleming JC, Haik BG (2004). Retained intraorbital metallic foreign bodies. Ophthalmic Plast Reconstr Surg.

[7] Vinodh VP, Sellamuthu P, Harun RH, Zenian MS (2014). Posterior intraorbital metallic foreign body: a case discussion. Med J Malaysia.

[8] Reyniers R, Paridaens D (2007). Spontaneous globe luxation and floppy eyelid syndrome in a patient with Hashimoto's disease. Eye (Lond).

[9] Anand S, Harvey R, Sandramouli S (2003). Accidental self-inflicted optic nerve head avulsion. Eye (Lond).

[10] Herman DC, Bartley GB, Walker RC (1987). The treatment of animal bite injuries of the eye and ocular adnexa. Ophthalmic Plast Reconstr Surg.

[11] Wu PS, Beres A, Tashjian DB, Moriarty KP (2011). Primary repair of facial dog bite injuries in children. Pediatr Emerg Care.

[12] Gurunluoglu R, Glasgow M, Arton J, Bronsert M (2014). Retrospective analysis of facial dog bite injuries at a Level I trauma center in the Denver metro area. J Trauma Acute Care Surg.

